# Mossy Fiber Sprouting in Temporal Lobe Epilepsy: The Impact of Netrin-1, DCC, and Gene Expression Changes

**DOI:** 10.3390/biomedicines12122869

**Published:** 2024-12-17

**Authors:** Melis Onay, Patrick N. Harter, Katherina Weber, Albrecht Piiper, Marcus Czabanka, Karl H. Plate, Thomas M. Freiman, Florian Gessler, Barbara Puhahn-Schmeiser

**Affiliations:** 1 Department of Internal Medicine I, Goethe University, Theodor-Stern-Kai 7, 60596 Frankfurt am Main, Germany; 2 Department of Neurosurgery, Rostock University Medical Center, Schilingallee 35, 18057 Rostock, Germany; 3 Department of Neuropathology, Ludwig-Maximillians-University Munich, Feodor-Lynen-Str. 23, 81377 Munich, Germany; 4 Department of Neuropathology (Edinger Institute), Goethe University, Heinrich-Hoffmann-Straße 7, 60528 Frankfurt am Main, Germany; 5 Department of Neurosurgery, Goethe University, Schleusenweg 16, 60528 Frankfurt am Main, Germany; 6 Department of Neurosurgery, Freiburg University Medical Center, Breisacher Str. 64, 79106 Freiburg, Germany

**Keywords:** temporal lobe epilepsy, hippocampal sclerosis, microglia, M1 phenotype, M2 phenotype, Netrin-1, DCC, synaptopodin, IκBα

## Abstract

Background: Temporal lobe epilepsy (TLE) is the most common form of drug-resistant epilepsy, often associated with hippocampal sclerosis (HS), which involves selective neuronal loss in the Cornu Ammonis subregion 1 CA1 and CA4 regions of the hippocampus. Granule cells show migration and mossy fiber sprouting, though the mechanisms remain unclear. Microglia play a role in neurogenesis and synaptic modulation, suggesting they may contribute to epilepsy. This study examines the role of microglia and axonal guidance molecules in neuronal reorganization in TLE. Methods: Nineteen hippocampal samples from patients with TLE undergoing epilepsy surgery were analyzed. Microglial activity (M1/M2-like microglia) and neuronal guidance molecules were assessed using microscopy and semi-automated techniques. Gene expression was evaluated using the nCounter Expression Profiling method. Results: Neuronal cell loss was correlated with decreased activity of the M1 microglial phenotype. In the CA2 region, neuronal preservation was linked to increased mossy fiber sprouting and microglial presence. Neuronal markers such as Deleted in Colorectal Cancer (DCC) and Synaptopodin were reduced in areas of cell death, while Netrin-1 was elevated in the granule cell layer, potentially influencing mossy fiber sprouting. The nCounter analysis revealed downregulation of genes involved in neuronal activity (e.g., NPAS4, BCL-2, GRIA1) and upregulation of IκB, indicating reduced neuroinflammation. Conclusions: This study suggests reduced neuroinflammation in areas of neuronal loss, while regions with preserved neurons showed mossy fiber sprouting associated with microglia, Netrin-1, and DCC.

## 1. Introduction

Epilepsy is recognized as one of the most prevalent neurological disorders, impacting around 50 million people globally [1] with a third of these cases exhibiting drug-resistant epilepsy. A common histopathological observation in such patients is temporal lobe epilepsy (TLE), which is often associated with hippocampal sclerosis (HS) [2,3]. Histological examinations reveal pronounced neuronal cell death across various hippocampal subregions [4], while the dentate gyrus (DG) exhibits limited cell loss. The neuronal cell death often occurs with interneuron loss and astrogliosis [5]. Moreover, secondary alterations such as granule cell dispersion (GCD) [6,7] and mossy fiber sprouting (MFS) have been described. Due to the loss of cells in CA3 and CA4, mossy fibers lose their target cells and sprout backwards into the granule cell layer and the inner molecular layer. The underlying mechanisms are not yet fully understood [8,9].

Possible triggers for this phenomenon include chronic inflammation and dysregulation of axonal guidance molecules. Yet the specific role of microglia in epilepsy remains unclear. Microglia are parenchymal macrophages of the central nervous system (CNS), known to regulate neurogenesis, phagocytosis, synapse formation, and brain function, and could play a critical role. Their activity is essential for maintaining synaptic function and brain homeostasis. Microglia polarize to different phenotypes, of which classically activated inflammatory microglia (microglia from M1-spectra) and alternatively immunosuppressive regenerative microglia (microglia from M2-spectra) represent two extremes of a dynamically changing activation state. The polarization of microglia is driven by signals from the tissue environment. Despite their importance, the exact roles of microglia and axonal guidance molecules in GCD and MFS are not well understood.

Previous studies have highlighted dual functions of the chemotropic guidance cue Netrin-1, which can act as either an attractive or repulsive cue, depending on receptor presence [10,11]. This duality suggests that Netrin-1 may play a significant role in (MFS).

The present study aims to investigate the roles of microglia and axonal guidance molecules in the pathophysiology of TLE, with a particular focus on their involvement in GCD and MFS. We hypothesized that alterations in microglial activity and the expression of axonal guidance molecules significantly contribute to the neuronal reorganization.

To test this hypothesis, we utilized immunohistochemical staining of hippocampal tissue from TLE patients, focusing on markers such as Iba-1, CD68, CD163, and CD31 to characterize microglial phenotypes. Additionally, the expression of neuronal guidance molecules, including Netrin-1 and its receptor Deleted in Colorectal Cancer (DCC), was analyzed to explore their potential roles in MFS. By integrating histological, molecular, and gene expression profiling approaches, this study seeks to address critical gaps in the understanding of hippocampal remodeling in TLE and to provide insights into the mechanisms driving neuronal reorganization in this condition.

## 2. Materials and Methods

### 2.1. Patient Selection

We included patients with drug-resistant TLE who underwent surgical intervention at the Department for Neurosurgery at the University Hospital Frankfurt. The resected tissues were subsequently examined at the Edinger Institute (Neuropathological Institute), Goethe University Frankfurt, Germany, a member of the Center for Personalized Translational Epilepsy Research (CePTER. The study was approved by the Ethics Committee of Goethe University Frankfurt, Germany (Approval ID: SNO-09-2014, 17 November 2014). A database for refractory epilepsy was established using paraffin-embedded tissues. The diagnosis adhered to the consensus statement of the International League Against Epilepsy [4]. Before initiating the study, we reviewed microscopic sections from all patients in our epilepsy database. We selected only hippocampi with distinct subregions, resulting in a total of 19 samples. To facilitate comprehensive comparison, we divided the hippocampi into two distinct groups: The first group consisted of TLE patients with a microscopically normal hippocampus, no hippocampal sclerosis (noHS), *n* = 10. The second group comprised patients afflicted by TLE accompanied by hippocampal sclerosis, hippocampal sclerosis (HS), *n* = 9.

### 2.2. Tissue Preparation and Immunohistochemistry

The brain tissues from the patients were fixed in 4% formalin (pH 7.4) and subsequently embedded in paraffin. Deparaffination and rehydration were carried out using standard alcohol and xylene procedures. Tissue sections, three micrometers thick, were obtained using a vibratome (Leica, Nussloch, Germany, Model Jung SM2000R). Immunohistochemistry (IHC) was conducted using the automated BOND 2 staining system (Leica Biosystems, Nussloch, Germany) in accordance with standard protocols. For the detection of microglial cell infiltration, the tissue sections were stained with the following antibodies: Iba-1 (FUJIFILM Wako Chemicals Europe, Neuss, Germany, Clone polyclonal, Dilution 1:1000), CD68 (Agilent, United States, Clone PG-M1, Dilution 1:200), CD163 (Novus Biologicals, Centennial, CO, United States, Clone 10D6, Dilution 1:500), CD74 (Abcam, Cambridge, UK, clone LN2, Dilution 1:100), and CD206 (Novus Biologicals, Centennial, CO, United States, Clone 5C11, dilution 1:3000). Additionally, hippocampal slices were stained with CD31 (Agilent, Santa Clara, CA, USA, Clone JC70A, Dilution 1:500) to explore potential correlations between microglial activation and enhanced angiogenesis. To investigate the relationship between neuronal cell death and microglia infiltration, the slices were also stained with anti-NeuN (Millipore, Darmstadt, Germany, Clone A60, dilution 1:4000). Neuronal plasticity assessment involved the use of neuronal markers such as Netrin-1 (Sigma Aldrich, Darmstadt, Germany, polyclonal, dilution 1:200) and DCC (Leica Biosystems, Nussloch, Germany, clone DM51, dilution 1:40). Additionally, Synaptopodin (R&D Systems, Minneapolis, United States, clone 44E3D12, dilution 1:50) and Synaptoporin (Synaptic Systems, Göttingen, Germany, clone 918842, dilution 1:700) were utilized.

### 2.3. Neuronal Cell Nuclei Counting

A semi-quantitative analysis was conducted to quantify neurons. The NeuN-stained slices were captured using an Axio ScanZ.1 (Carl Zeiss Microscopy GmbH, Jena, Germany) equipped with a 20× objective lens. The captured slides were uploaded in gzi file format into the image analysis software HALOTM Next-Generation (Version 3.0.311.287, Indica Labs, Albuquerque, NM, USA). Specialized software tools were employed to eliminate artifacts and larger vessels. Following manual selection of an area, the software automatically counted the neurons. Subsequently, the area was standardized to one square millimeter. Additionally, the ratio (number of neurons per square millimeter) was generated for comparative analysis between sclerotic and non-sclerotic hippocampi.

### 2.4. Microglial Density Analysis

The analysis of stained slices was conducted using a microscope (BX41, Olympus, Tokyo, Japan), with the images captured using an attached camera (Olympus, Tokyo, Japan). The quantification of activated microglia involved a manual approach. A small area was selected, and the microglial cells within it were enumerated. The dimensions of the areas were then standardized to one square millimeter. Subsequently, a ratio (number of activated microglia cells/square millimeter) was established for comparison between sclerotic and non-sclerotic hippocampi. Furthermore, a comparative analysis was performed among different subregions within the hippocampi [12].

### 2.5. Neuronal Cell Density Analysis

Neuronal density analysis was carried out utilizing a digital semi-automated quantification approach. Stained hippocampal slices were captured using an Axio ScanZ.1 microscope (Carl Zeiss Microscopy GmbH, Jena, Germany) equipped with a 20× objective lens. Subsequently, the captured slices were uploaded in gzi file format to the HALOTM Next-Generation Image Analysis software (Version 3.0.311.287, Indica Labs, Albuquerque, NM, USA). Software tools were applied to remove larger vessels and artifacts. The program possesses the capability to discern distinctive distributions and various staining intensities. A designated area was analyzed and further subdivided into stained sections. This approach aimed to identify potential differences in the expression of neuronal markers within the cohorts. To facilitate comparison, a ratio (area with positive stained tissue/total tissue area) was established, allowing for comparisons between groups and among distinct hippocampal subregions [13].

### 2.6. RNA and Protein Isolation

Initially, all hippocampi from FFPE samples underwent haematoxylin and eosin staining to identify specimens displaying all regions of interest. Subsequently, we selected the suitable hippocampi, resulting in a total of six human specimens per group. Tissue sections measuring three micrometers were obtained from these specimens. RNA extraction was conducted using the RNA Kit (Covaris, Woburn, MA, USA) in accordance with the manufacturer’s protocol.

The protein isolation involved deparaffinization followed by heat-induced antigen retrieval to reverse formalin crosslinking. The proteins were extracted using a specialized lysis buffer containing detergents and protease inhibitors to solubilize the proteins while preserving their integrity (RIPA Lysis Buffer System, Santa Cruz Biotechnologies, Heidelberg, Germany). The extracted proteins were then quantified and prepared for subsequent analysis with the NanoString nCounter system.

### 2.7. NCounter Expression Profiling

The nCounter assay offers a single-tube, highly sensitive, and reproducible approach for detecting various nucleic acid targets. These assays directly detect targets using molecular barcodes, eliminating the need for reverse transcription or amplification. Processing of nCounter assays was automated at the Prep Station, followed by data collection on a Digital Analyzer. The obtained data can be analyzed and prepared for statistical analyses using the nSolver 4.0 Software Analysis System [14]. This analysis was conducted in collaboration with NanoString Technologies (Seattle, WA, USA) and the Department of Pathology of the Goethe University in Frankfurt.

We used the neuropathology panel, which includes more than 700 relevant genes and proteins that have already been described in the context of neuropathological disease. The characteristic genes for each cell type are summarized in the supplementary data (Supplementary Data, Table S1).

### 2.8. Statistical Analysis

The statistical analysis was carried out using Prism (Version 10.0.2 (171)) and the nSolver Software Analysis System (Version 4.0).

The quantification of various cell type scores was accomplished using Qc plots. Qc plots allow for the quantification of cell type abundance by taking the logarithm of the gene expression specific to each cell type. For the statistical analysis, principal component analysis was used. It involved data preparation, centering, calculating the covariance matrix, eigenvalue decomposition, selecting principal components, and transforming the data.

## 3. Results

### 3.1. Neuronal Cell Loss Correlates with a Decreased Microglial Activty of the M1 Phenotype

**Figure 1:**
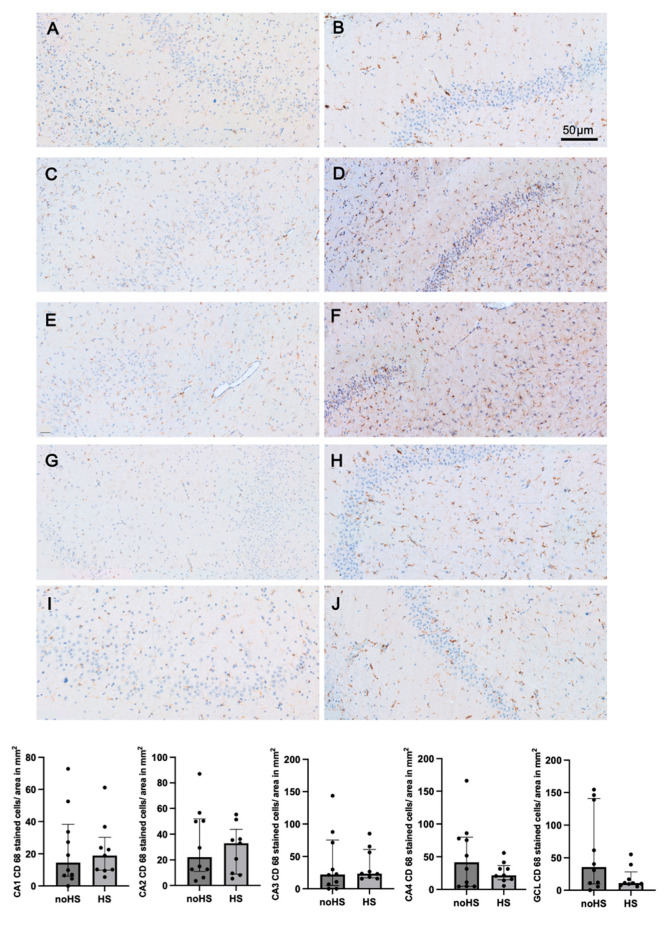
Subregions of healthy hippocampi (noHS) and sclerotic hippocampi (HS) stained with CD68. Activated microglia is recognizable by thickened and retracted branches. In the bar diagrams, bars and error bars indicate medians and IQR. (**A**) CA1 subregion of noHS. (**B**) CA1 subregion of HS. (**C**) CA2 subregion of noHS. (**D**) CA2 subregion of HS—here you can detect a significant overexpression of Iba-1. (**E**) CA3 subregion of noHS. (**F**) CA3 subregion of HS. (**G**) CA4 subregion of noHS. (**H**) CA4 subregion of HS. (**I**) Granule cell layer of noHS. (**J**) Granule cell layer of HS.

Our investigation revealed elevated levels of Iba-1, a marker for microglia and macrophages, particularly prominent in hippocampal sclerosis. Notably, significant expression was observed in the CA2 subregion (Supplementary Data, Figure S1). CD163 and CD206, markers of M2-like microglia, exhibited elevated levels in almost all subregions of the sclerotic hippocampus. CD163 displayed a significant rise in the CA1 subregion (Supplementary Data, Figure S2), whereas CD206 showed increased expression in the CA3 and CA4 subregions (Supplementary Data, Figure S3). There was no evidence for differences in the levels of CD68, a marker for M1-like microglia, between pathological and non-sclerotic hippocampi (Figure 1). CD74, another marker for M1-like microglia, also showed no significant differences. However, the non-sclerotic hippocampi demonstrated slightly elevated levels of activated microglial activity (Supplementary Data, Figure S4). CD31, a marker for blood vessels, did not present significant differences between hippocampal sclerosis and non-sclerotic tissue. However, there was a trend towards an increased CD31 expression in pathological tissue (Supplementary Data, Figure S5).

Together, these data suggest a potential correlation between hippocampal cell loss and reduced microglial activity.

### 3.2. Epileptic Conditions Cause a Shift in Expression of Neuronal Markers Inducing Mossy Fiber Sprouting

**Figure 2:**
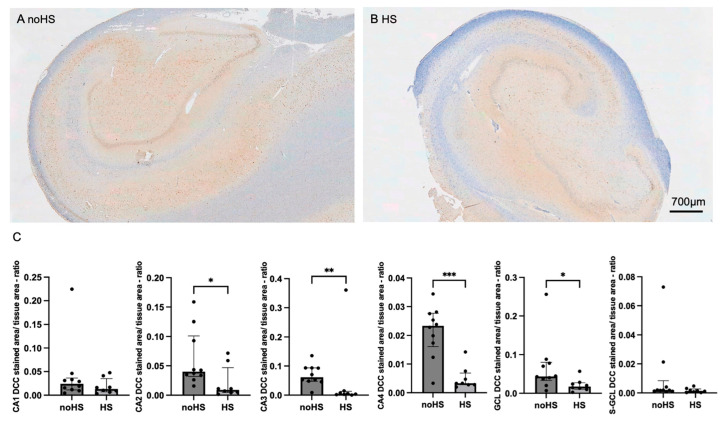
The levels of DCC in noHS and HS hippocampal slices. (**A**) Expression of DCC in noHS. (**B**) Expression of DCC in HS revealing a loss of DCC in all subregions. (**C**) One-factorial analysis of DCC-positive stained area/tissue area mm^2^ in HS and noHS. The semi-automatized analysis shows a significant downregulation of DCC in subregion CA2, CA3, CA4, and GCL in the sclerotic tissue. Bars and error bars indicate medians and IQR. * *p* < 0.05; ** *p* < 0.01; *** *p* < 0.001.

**Figure 3:**
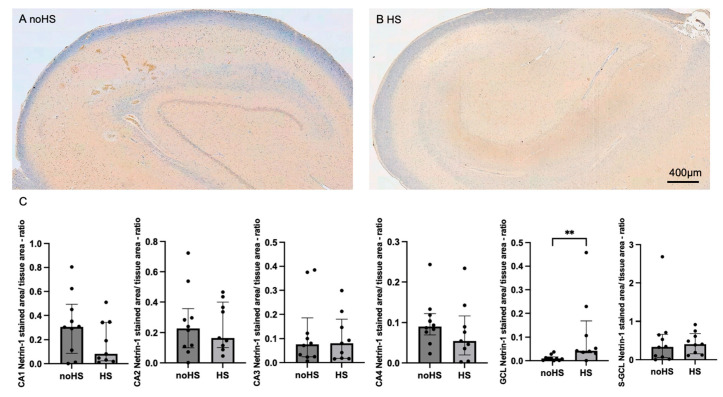
IHC staining of Netrin-1 in noHS and HS in hippocampal slices. (**A**) Expression of Netrin-1 in noHS (**B**) and in HS, displaying a loss of Netrin-1 in almost all subregions. (**C**) One-factorial analysis of Netrin-1-positive area/tissue area mm^2^ in HS. A significant upregulation is detectable in the GCL region of HS. Bars and error bars indicate medians and IQR. ** *p* < 0.01.

Immunohistochemical stainings of the hippocampi revealed lower expression of synaptopodin in sclerotic tissue, with significant differences observed in the CA1, CA2, CA3, and CA4 subregions (Supplementary Data, Figure S6). Synaptoporin also demonstrated significantly decreased expression in the CA1, CA2, and CA4 subregions of sclerotic hippocampi (Supplementary Data, Figure S7). Although we observed a downregulation of synaptoporin in the sclerotic tissue, this downregulation was less pronounced in the CA2 region. This might be due to reduced cell death. Of particular significance, the DCC exhibited elevated levels primarily in the CA1, CA2, CA3, and CA4 subregions of non-sclerotic hippocampi (Figure 2). Interestingly, Netrin-1 showed higher levels in the sclerosis-affected subregions CA3, GCL, and S-GCL (Figure 3).

### 3.3. Epileptic Conditions Lead to Variable Changes in Gene Expression Profiles (nCounter Expression Profiling) and Influence Neural Activity

**Figure 4:**
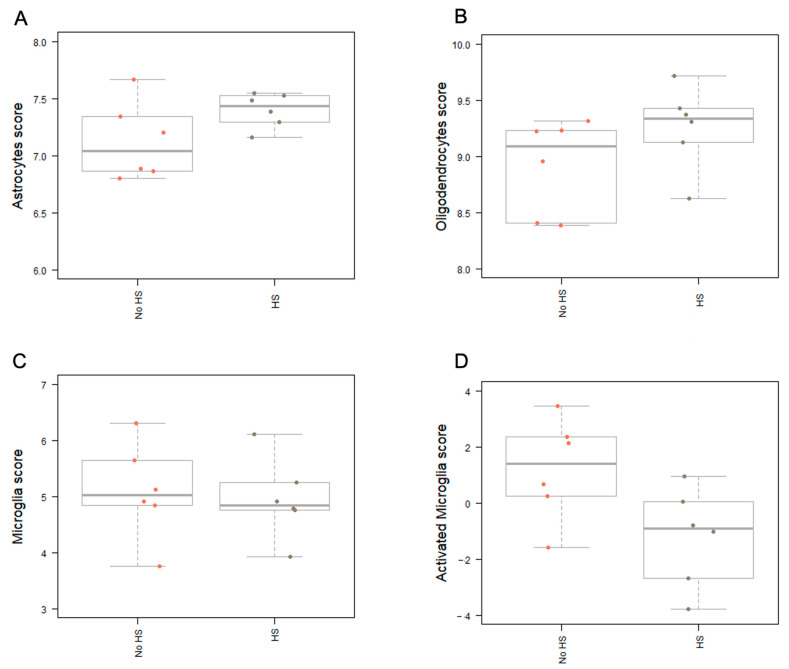
Cell type analyses according to the expression of different cell type scores in noHS and HS. For the investigation, six samples were used per group. Each point represents a sample. Boxes, bars and error bars indicate medians and IQR. The score is calculated from the cell type abundances by taking the logarithm of the expression of the genes specific to the cell types. (**A**) Astrocytes score (**B**) Oligodendrocytes score—An increased oligodendrocytes score (genes that occur in oligodendrocytes) is detectable in the sclerosis but there are no significant differences. (**C**) Microglia score—Surprisingly, an increased number of genes encoding for macrophages in general is apparent, even though the results are not significant. (**D**) Activated microglia score—The genes specifically associated with activated microglia exhibit higher expression in the noHS group, although without a significant result.

Gene expression analyses of the human samples revealed a trend of an upregulation of oligodendrocytes and astrocytes in hippocampal sclerosis (Figure 4). Intriguingly, the expression of microglia and activated microglia markers tended to be lower in sclerotic tissue (Figure 4).

The levels of mRNAs characteristic of axons, dendritic structures, neuronal connectivity, transmitter synthesis, and uptake were markedly lower in hippocampal sclerosis (Supplementary Data, Figure S6). In contrast, myelination and autophagy are upregulated in sclerotic tissue, reflecting a response to the remnants of cell death.

**Figure 5:**
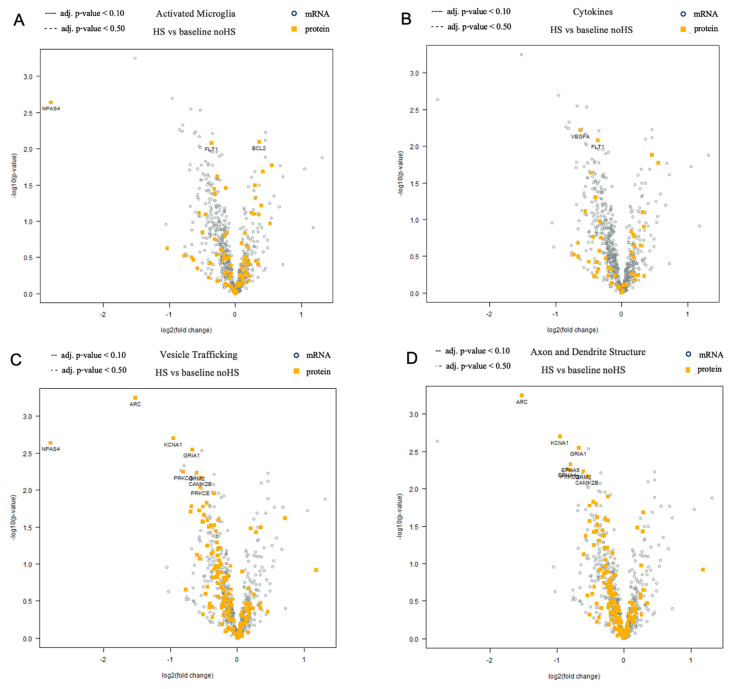
Differential levels of mRNAs and proteins between non-sclerotic (noHS) and sclerotic tissue (HS) are presented in volcano plots. (**A**) Differential levels of mRNAs and proteins involved in activated microglia. The largest differences were seen in the proteins NPAS4, FLT1, and BCL2, but none reached statistical significance. (**B**) Differential levels of cytokine mRNAs and proteins in HS and noHS. The highest differences were seen in VEGFA and FLT1, which, however, did not reach statistical significance. (**C**) Differential levels of mRNAs and proteins involved in vesicle trafficking. The highest differences are observed in NPAS4, ARC, KCNA1, and GRIA1; however, this did not reach statistical significance. (**D**) Differential levels of mRNAs involved in axon and dendrite structure in HS and noHS. The highest differences were seen in ARC, KCNA1, and GRIA1, again without reaching statistical significance.

In addition, we identified differences in the gene expression patterns between sclerotic and non-sclerotic tissue. In the gene expression pattern of microglia and cytokines, it was observed that the NPAS4 was upregulated in HS (Figure 5). Additionally, FLT-1 and VEGF proteins exhibit downregulation in sclerotic tissue, but this did not reach statistical significance (Figure 5).

Conversely, our analyses indicated an increase in the BCL2 in HS (Figure 5). The protein ARC appeared to be downregulated in the sclerotic tissue (Figure 5). Likewise, levels of the protein KCNA and GRIA appeared to be diminished in the HS (Figure 5).

**Figure 6:**
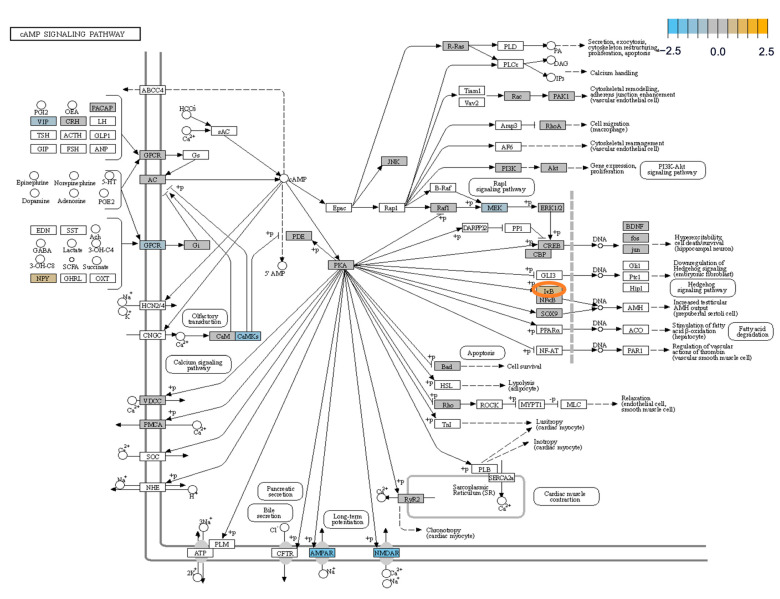
Exemplary scheme of the expression of signaling influenced by cAMP in HS. The pathway was created by the summarization of pathway scores, which were calculated on the principal component analysis of the pathway genes’ normalized expression (refer to the Section 2 for details). The mRNAs, which were found to be downregulated are blue colored, whereas upregulated mRNAs are gold colored. Interestingly, the expression of IκB was upregulated, which, in turn, leads to reduced activity of NFκB, as the NFκB pathway is inhibited by IκB. This could be interpreted as a sign of reduced neuroinflammation.

The analyses of the pathways regulated by cAMP showed that IκBα (nuclear factor of kappa light polypeptide gene enhancer in B-cells inhibitor alpha) is overexpressed in HS (Figure 6).

## 4. Discussion

A substantial body of research has focused on the pro-epileptogenic effects of M1-like microglia. Our findings, however, indicate a reduction in activated microglia within hippocampal sclerosis, confirmed by microglia density and nCounter analyses. This suggests that microglia may have neuroprotective or even anti-epileptic functions. This idea is supported by a study demonstrating that pharmacological depletion of microglia worsens cell loss [15].

Our data also show an upregulation of IκB in HS, leading to the suppression of NFkB and neuroinflammation. Oligodendrocytes and astrocytes were more prevalent in sclerotic tissue, indicative of the aftermath of extensive cell death. Additionally, a recent study has also introduced the concept of “gliosis only” in non-sclerotic hippocampi of TLE patients [11]. These hippocampi display elevated gliosis and microglia without prominent neuronal cell death. The increased presence of microglia might contribute to more severe seizures and poorer surgical outcomes. This may explain the elevated activated microglia and neuroinflammation observed in the non-sclerotic tissue during the late chronic phase. However, it is important to acknowledge that epilepsy occurs across various stages. Understanding the role of microglia and other factors in the initial phase of epilepsy is crucial for a comprehensive understanding of the disease progression. Emerging evidence suggests that microglia and neuroinflammation may exert a significant influence on tissue remodeling during the initial phases of epilepsy [16,17,18,19].

Another interesting aspect of our microglia analyses is the findings within the CA2 subregion. Although the results are not statistically significant, the number of microglia appeared to be elevated. This might be related to the relatively small loss of neurons in this subregion. A previous study has also highlighted the CA2 subregion, where an enhanced mossy fiber sprouting was observed [20], which could be associated with a relatively low neuronal cell death. In future studies, it is essential to investigate the function of microglia in different stages of epilepsy and in individual subregions to gain a detailed understanding of the pathogenesis.

In addition to significant cell death, MFS is another pathology, representing hippocampal sclerosis. In healthy tissue, MF innervate the pyramidal cells and interneurons in the CA3 subregion. Given the pivotal role of CA3 in the hippocampus, the loss of these cell types in epileptic conditions prompts MF to retract [8,9]. A study involving Netrin-1 is documented in this context. Netrin-1 is a bifunctional protein, exhibiting both attractive and repulsive properties [11]. The function of Netrin-1 depends on the underlying molecule. In healthy tissue, Netrin-1 and DCC are presumed to have an attractive effect in the MF in the CA3 subregion [21]. In our study, we showed that Netrin-1 was reduced in all areas within the sclerotic tissue, and DCC was significantly downregulated in the CA1 to CA4 subregions. This pathology appears to negatively influence neuronal plasticity and promote the progression of the disease. This hypothesis warrants further validation through rigorous investigation. Netrin-1 could be a promising pharmacological approach.

Synaptopodin is an actin-associated protein which plays a role in the structural plasticity of dendritic spines. It is involved in the stabilization and enlargement of dendritic spines, being important for the formation and maintenance of synapses. A previous study described a notable decrease of Synaptopodin following a status epilepticus [22]. This observation aligns with our finding of a reduced level of Synaptopodin in the subregions CA1-CA4. This loss should lead to the demise of synapses and, over time, hippocampal sclerosis. However, the exact trigger that leads to a loss of Synaptopodin in the subregions CA1-CA4 remains unclear.

A unique aspect of our study lies in nCounter sequencing of human samples, contrasting with previous analyses mostly conducted on rodent tissue. Recognizing the divergence between human and rodent genomes, these results corroborate our IHC findings. Notably, most factors showed downregulation, like the activated microglia, apoptosis, neuronal connectivity, and the transmitter release. However, astrocytes, oligodendrocytes, and autophagy were elevated in the hippocampus. Another interesting aspect of the present study is the analysis of the cAMP signaling pathway in epilepsy. The cAMP signaling pathway has been investigated in several studies, and cAMP-induced hyperexcitability has already been linked to the development of epilepsy [23,24,25]. Suppression of CREB in mouse models shortened the duration of the status and reduced the number of epileptic seizures [26]. The long-term effect of cAMP operates through the cAMP-PKA-CREB transcriptional signaling in epilepsy. This pathway is associated with MFS, seizure arising and spontaneous seizures [27]. However, we were unable to confirm this result. Nonetheless, our data suggest that IκB was increased, suppressing neuroinflammation. This could be a regulatory countermeasure. However, it is possible that in the late phase of epilepsy, the pathological alterations have happened and the upregulation of I-κB is a remnant. A better understanding of the influence of cAMP on the development of epilepsy is necessary.

Our nCounter analysis has unveiled intriguing insights, identifying genes expressed differentially between HS and noHS, like *npas4*, *bcl-2*, *gria1*, and *kcna*. *npas*4 is a protein-coding gene that plays a role in regulating the expression of other genes in response to neuronal activity. It is associated with synaptic plasticity—the ability of neurons to form and modify connections between each other in response to electric stimulation [28,29]. The dysregulation of this gene has been implicated in various neurological and psychiatric disorders [30,31]. In this context, research is being conducted with a therapeutic possibility of influencing the expression of this gene. This could also represent a therapeutic opportunity in hippocampal sclerosis, as neuronal plasticity is altered by epilepsy. The function of *npas4* in epileptogenesis was investigated in a rodent model [32]. It seems that *npas4* has an inhibitory effect.

The BCL2 protein is an anti-apoptotic protein that functions to promote cell survival by preventing apoptotic pathways. Dysregulation of the *bcl2* gene and overexpression of the BCL2 protein have been implicated in the development and progression of various cancers. BCL2 has been associated with a neuroprotective property in relation to epilepsy. The upregulation may be a countermeasure to prevent cell death. In a mouse model of seizure-induced neuronal cell death, the viral transfection of BCL-2 seemed to have a positive effect as well [33,34]. The regulation of BCL2 may be a new pharmacological target for therapy-resistant epilepsy.

The protein KCNA refers to the potassium voltage-gated channel subfamily A member 1 Gene (KCNA1). This channel is important for regulating the electrical activity of neurons. The role of KCNA1 in epilepsy has been studied using mouse models. The findings suggest that KCNA1 mutations may contribute to the development and progression of epilepsy [35,36]. GRIA1 is encoded by the gene glutamate ionotropic receptor AMPA type subunit 1, and is important for regulating synaptic plasticity. The role of GRIA1 in epilepsy has been studied as well. It has been shown that GRIA1 expression is altered in the brains of patients with epilepsy and in animal models of epilepsy [37,38]. Reducing the expression of GRIA1 seemed to reduce the severity of seizures and to decrease the likelihood of epileptogenesis [37]. Our results indicate a downregulation of both GRIA1 and KCNA1 in HS. This downregulation might be attributed to chronic inflammation in epilepsy. Another plausible explanation is that these genes exhibit increased expression in non-sclerotic tissue, contributing to a negative disease progression. Patients with TLE without hippocampal sclerosis often experience more severe seizures and have poorer surgical outcomes. The complexity of these gene dynamics and their impact on epilepsy outcomes underscores the need for further scientific exploration to uncover more satisfactory answers.

One limitation of our study is the focus on the late chronic phase of epilepsy, which may restrict the generalization of our findings to earlier stages of epileptogenesis. Additionally, the relatively small sample size limits the statistical power and generalizability of our results. Variability among patient samples, such as differences in age, sex, disease severity, or treatment history, might also contribute to variations in the observed outcomes. Furthermore, the use of human tissue samples from surgical interventions introduces potential biases, as these samples may not fully represent the broader spectrum of TLE pathology, particularly in the earlier stages. Despite these limitations, we also acknowledge the lack of statistical significance in certain observations, such as the downregulation of specific genes. This underscores the need for cautious interpretation of our findings. Larger sample sizes and further studies are needed to validate these results and establish their broader significance.

## 5. Conclusions

Our study advances understandings of temporal lobe epilepsy (TLE) by uncovering the complex roles of microglia. It suggests their potential neuroprotective effects and highlights the significance of neuroinflammation and gene expression changes in the pathogenesis of epilepsy. We identified a reduction in activated M1 microglia and an upregulation of IκB in sclerotic tissue, pointing to a nuanced interplay between microglial activity and neuroinflammation. Furthermore, MFS seems to be associated with a loss of axonal guidance molecules like Netrin-1 and DCC. The extent to which the upregulation of these molecules contributes to an improvement in this remodeling process is a subject for future evaluation. Our gene expression analysis revealed critical insights into the cellular response to epilepsy. This included altered expression of oligodendrocytes, astrocytes, and key neuronal genes, in particular *npas4*, which underscores the disease’s impact on neuronal connectivity and plasticity. Future research should focus on the multifaceted molecular landscape of TLE, particularly microglia’s role across epilepsy stages and the potential of gene therapies to mitigate hippocampal sclerosis and epileptogenesis.

## Data Availability

The data presented in this study are available upon request from the corresponding author, given the ethics committee approval to execute the study does not apply to making raw data and related clinical and demographic information publicly available.

## Supplementary Materials

The following supporting information can be downloaded at: https://www.mdpi.com/article/10.3390/biomedicines12122869/s1, Figure S1: Subregions of noHS and HS stained with Iba-1, a panmarker for activated Microglia. Iba-1 is slightly more expressed in the sclerotic tissue. (**A**) CA1 subregion of noHS (**B**) CA1 subregion of HS (**C**) CA2 subregion of noHS (**D**) CA2 subregion of HS (**E**) CA3 subregion of noHS (**F**) CA3 subregion of HS (**G**) CA4 subregion of noHS (**H**) CA4 subregion of HS (**I**) Granule cell layer of noHS (**J**) Granule cell Layer of HS (**K**) One-factorial analysis of Iba-1 positive stained cells/surface mm^2^ in noHS and HS. Error bars indicate IQR. Iba-1 is slightly more expressed in the sclerotic tissue. A significant higher expression is detectable in the CA2 subregion of the sclerosis. * *p* < 0.05; ** *p* <0.01; *** *p* <0.001; Figure S2: One-factorial analysis of CD163 positive stained cells/surface mm^2^ in noHS and HS. Error bars indicate IQR. CD163 is a marker for M2 Microglia. It is slightly more expressed in healthy tissue with a significant difference in the CA1 subregion of the sclerosis. * *p* <0.05; ** *p* <0.01; *** *p* <0.001; Figure S3: One-factorial analysis of CD206 positive stained cells/surface mm^2^ in noHS and HS. Error bars indicate IQR. CD206 is a marker for M2 Microglia. It is slightly more expressed in healthy tissue with a significance in the CA3 and CA4 subregions of the sclerosis. * *p* < 0.05; ** *p* < 0.01; *** *P* < 0.001; Figure S4: The Expression of Deleted in Synaptopodin (Podin) in noHS and HS. Error bars indicate IQR. (**A**) The expression of Podin in noHS (**B**) The expression of Podin in HS, where you can see a detectable loss of Podin in all subregions. (**C**) One-factorial analysis of Podin positive stained area/tissue area mm^2^ in HS and noHS. The semi-automatized analysis shows a significant downregulation of Podin in the subregions CA1, CA2, CA3 and CA4 in the sclerotic tissue. * *p* <0.05; ** *p* < 0.01; *** *p* < 0.001, **** *p* < 0.0001; Figure S5: The Expression of Deleted in Synaptoporin (Porin) in noHS and sclerotic tissue HS. Error bars indicate IQR. (**A**) The expression of Porin in noHS (**B**) The expression of Porin in HS, where you can see a detectable loss of Porin in all subregions. (**C**) One-factorial analysis of Porin positive stained area/tissue area mm^2^ in HS and noHS. The semi-automized analysis shows a significant downregulation of Porin in the subregions CA1, CA2, CA3 and CA4 in the sclerotic tissue. * *p* < 0.05; ** *p* < 0.01; *** *p* < 0.001; Figure S6: The Expression of different Cell Types in noHS and sclerotic tissue HS. For the investigation, six samples were used per group. Each point represents a sample. The score is calculated from the cell type abundances by taking the logarithm of the expression of the genes specific to the cell types. (**A**) Apoptosis score—A decreased apoptosis score is present in the sclerosis without any significant differences. (**B**) Autophagy score—A decreased oligodendrocytes score is detectable in the sclerosis. However, there are no significant differences. (**C**) Neuronal connectivity score—As expected, this score is higher in noHS. But the difference is not statistically significant. (**D**) Transmitter release score—The transmitter release score is increased in noHS since there is no neuronal cell death. The difference is not significant; Table S1: Overview of the main genes used in the NaNo-String analysis to characterize the individual cell types. Subregions of noHS and HS stained with Iba-1, a panmarker for activated Microglia. Iba-1 is slightly more expressed in the sclerotic tissue.
