# Supplementary material for: Mossy Fiber Sprouting in Temporal Lobe Epilepsy: The Impact of Netrin-1, DCC, and Gene Expression Changes
Source: Biomedicines. 2024 Dec 17;12(12):2869. doi: 10.3390/biomedicines12122869 (PMC11672915; doi:10.3390/biomedicines12122869)
Supplement: Supplementary file 1 [file biomedicines-12-02869-s001.zip › biomedicines-3341933-supplementary.pdf]

Supplementary Data

Cell Type	Associated human genes
**Neurons**	DLX1, DLX2, GRM2, ISLR2, SLC17A6, TBR1
**Astrocytes**	ALDH1L1, EGFR, ENTPD2, GDPD2, ITGA7, KIAA1161, NWD1, SOX9
**Microglia**	GPR84, IRF8, LRRC25, NCF1, TLR2, TNF, AIF1, TMEM119, ITGAM, CX3CR1, P2RY12, SPI1
**Oligodendrocytes**	BCAS1, ERBB3, FA2H, GAL3ST1, GJB1, GSN, MYRF, NINJ2, PLLP, PLXNB3, PRKCQ, SOX10, UGT8
**Endothelial Cells**	CLDN5, EMCN, ESAM, FLT1, ICAM2, LSR, MYCT1, NOSTRIN, TIE1

Table S1 – Overview of the main genes used in the NaNo-String analysis to characterize the individual cell types. - Subregions of noHS and HS stained with Iba-1, a panmarker for activated Microglia. Iba-1 is slightly more expressed in the sclerotic tissue

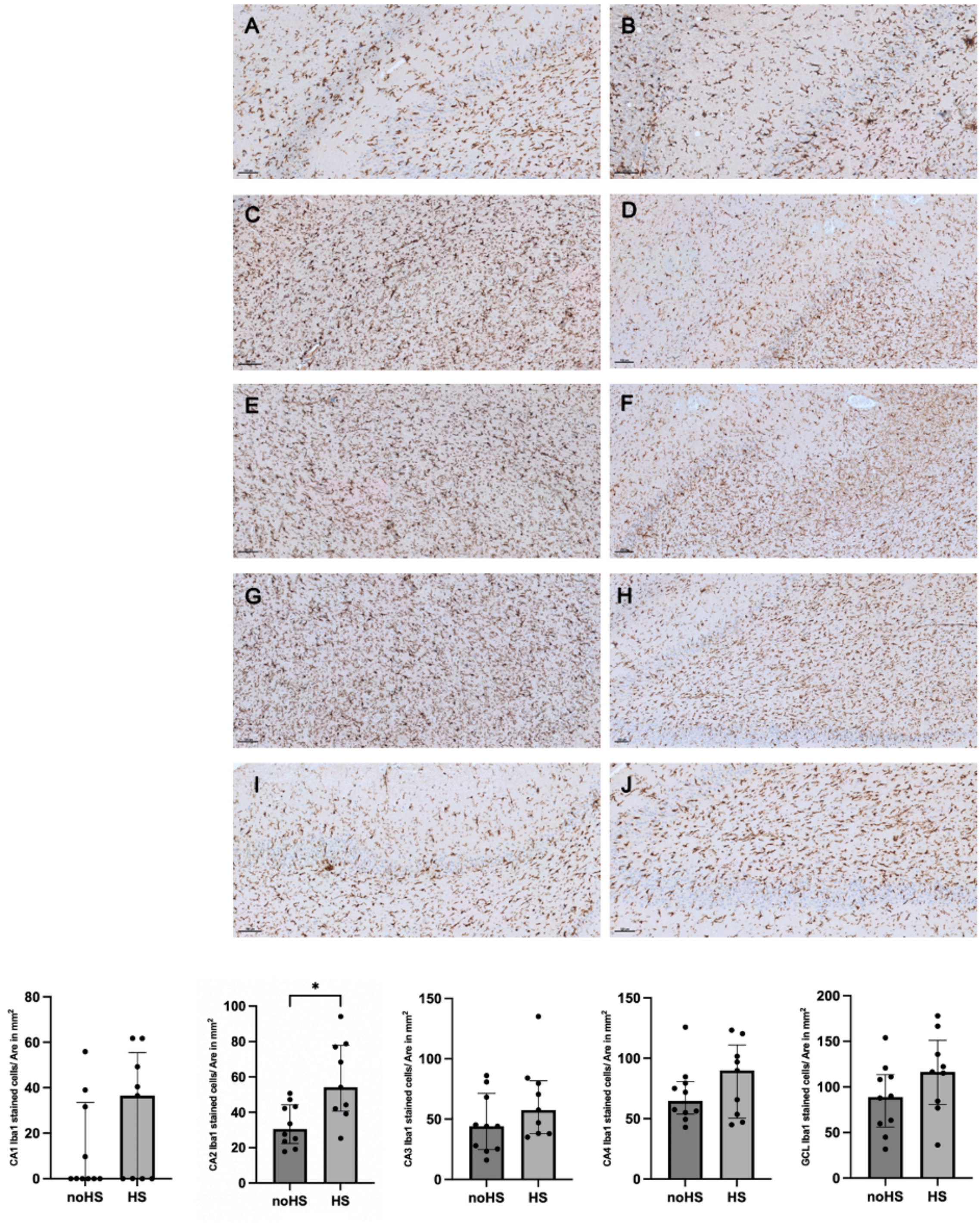

Figure S1- Subregions of noHS and HS stained with Iba-1, a panmarker for activated Microglia. Iba-1 is slightly more expressed in the sclerotic tissue.
(A) CA1 subregion of noHS (B) CA1 subregion of HS
(C) CA2 subregion of noHS (D) CA2 subregion of HS
(E) CA3 subregion of noHS (F) CA3 subregion of HS
(G) CA4 subregion of noHS (H) CA4 subregion of HS
(I) Granule cell layer of noHS (J) Granule cell Layer of HS
(K) One-factorial analysis of Iba-1 positive stained cells/ surface mm^2 in noHS and HS. Error bars indicate IQR. Iba-1 is slightly more expressed in the sclerotic tissue. A significant higher expression is detectable in the CA2 subregion of the sclerosis. *P<0.05; **P<0.01; ***P<0.001.

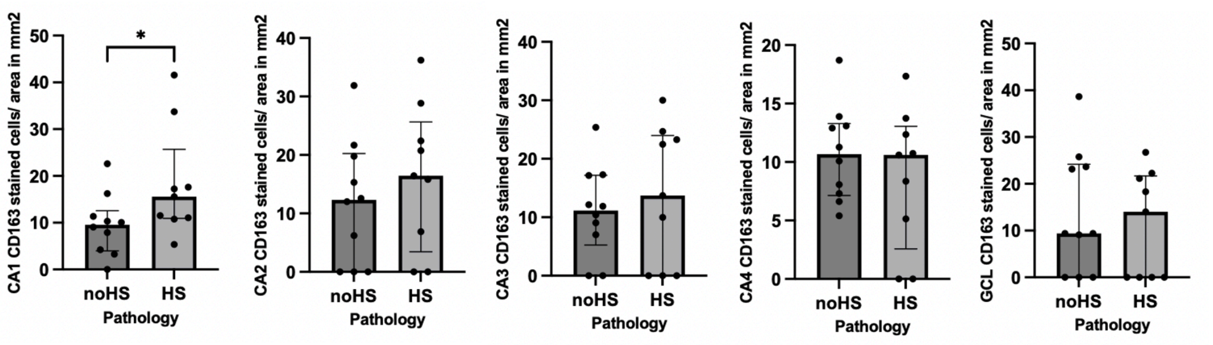

Figure S2- One-factorial analysis of CD163 positive stained cells/ surface mm^2 in noHS and HS. Error bars indicate IQR.
CD163 is a marker for M2 Microglia. It is slightly more expressed in healthy tissue with a significant difference in the CA1 subregion of the sclerosis.
*P<0.05; **P<0.01; ***P<0.001.

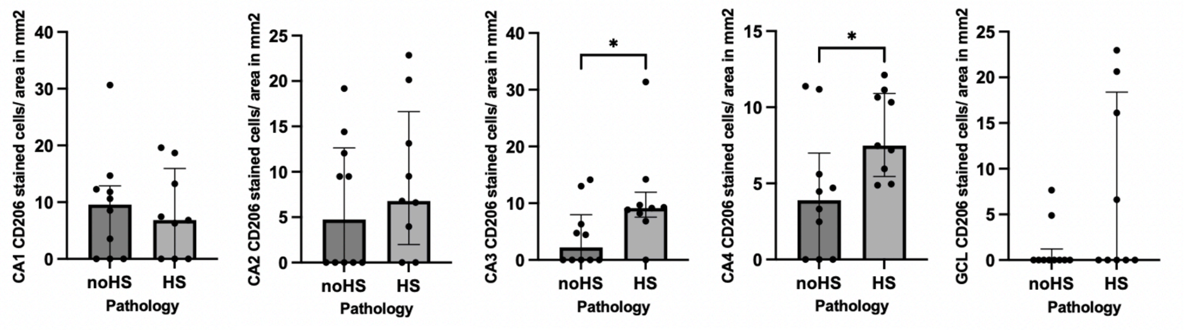

Figure S3- One-factorial analysis of CD206 positive stained cells/ surface mm^2 in noHS and HS. Error bars indicate IQR.
CD206 is a marker for M2 Microglia. It is slightly more expressed in healthy tissue with a significance in the CA3 and CA4 subregions of the sclerosis.
*P<0.05; **P<0.01; ***P<0.001.

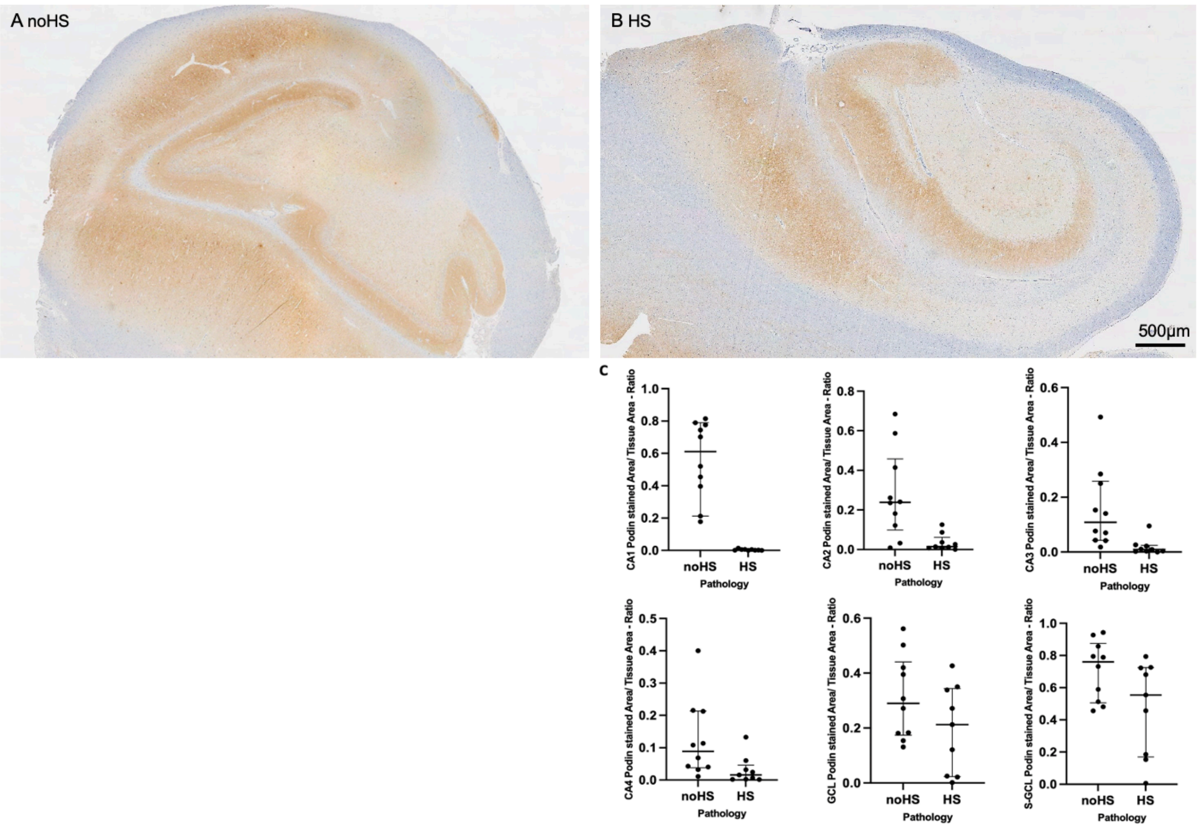

Figure S4– The Expression of Deleted in Synaptopodin (Podin) in noHS and HS. Error bars indicate IQR.
(**A)** The expression of Podin in noHS (**B**) The expression of Podin in HS, where you can see a detectable loss of Podin in all subregions. (**C**) One-factorial analysis of Podin positive stained area/ tissue area mm² in HS and noHS. The semi-automatized analysis shows a significant downregulation of Podin in the subregions CA1, CA2, CA3 and CA4 in the sclerotic tissue. *P<0.05; **P<0.01; ***P<0.001, ****P<0.0001.

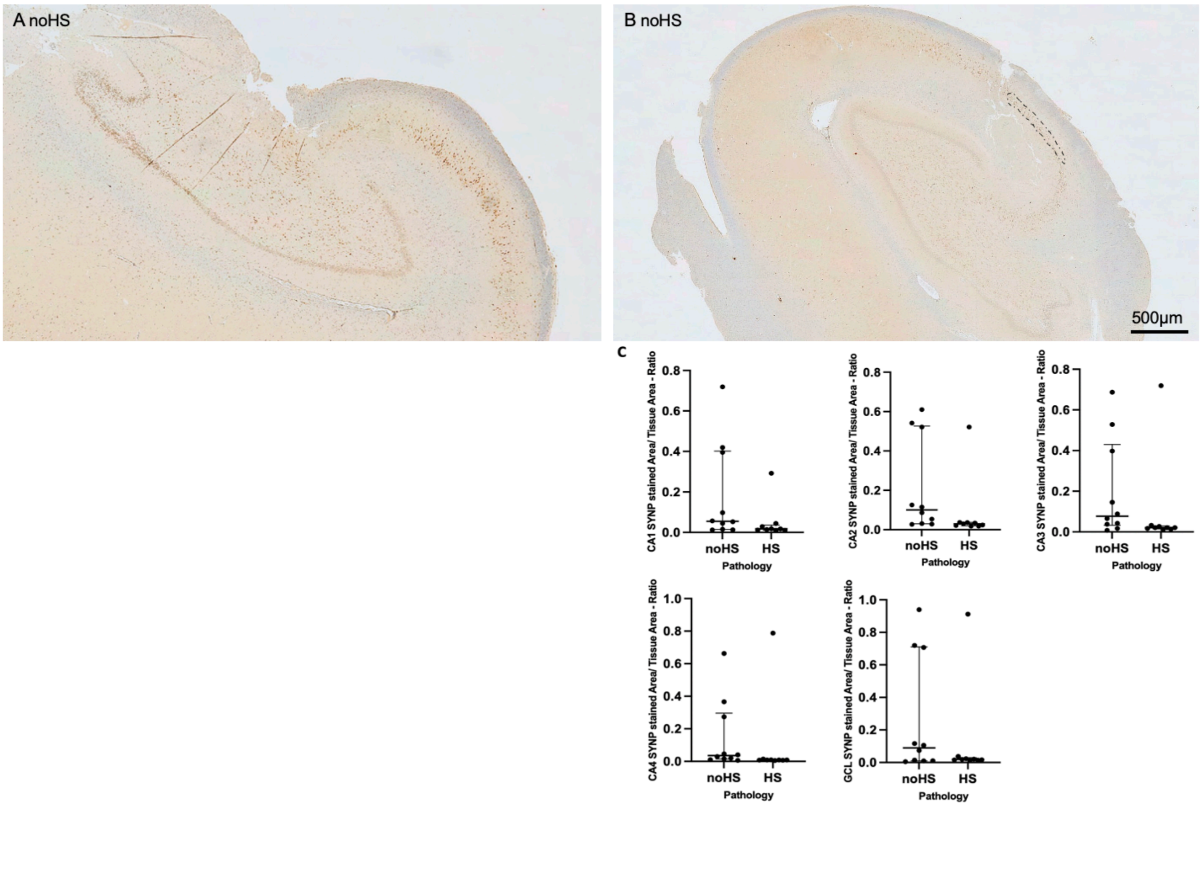

Figure S5– The Expression of Deleted in Synaptoporin (Porin) in noHS and sclerotic tissue HS. Error bars indicate IQR.
(**A)** The expression of Porin in noHS (**B**) The expression of Porin in HS, where you can see a detectable loss of Porin in all subregions. (**C**) One-factorial analysis of Porin positive stained area/ tissue area mm² in HS and noHS. The semi-automized analysis shows a significant downregulation of Porin in the subregions CA1, CA2, CA3 and CA4 in the sclerotic tissue. *P<0.05; **P<0.01; ***P<0.001.

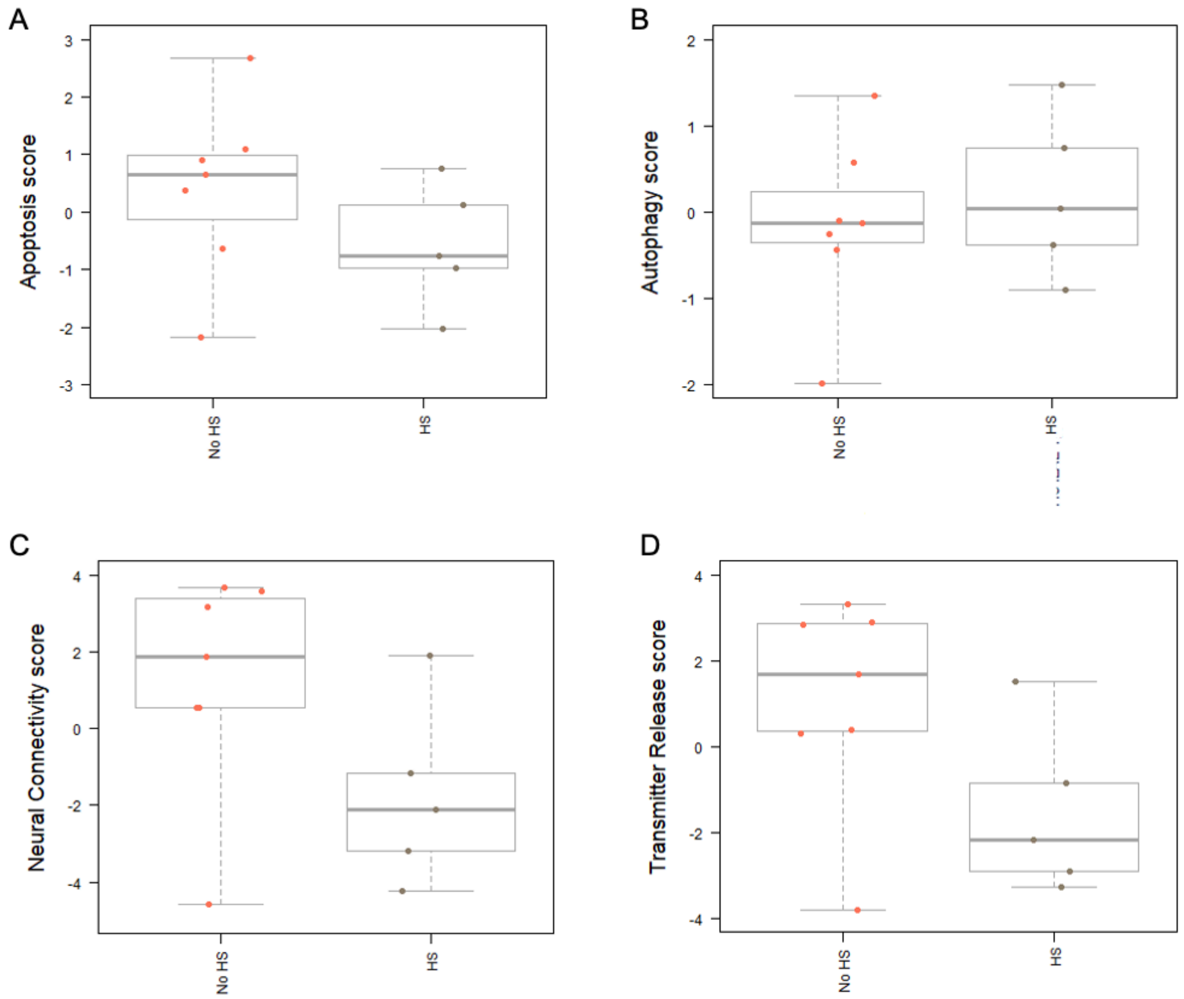

Figure S6- The Expression of different Cell Types in noHS and sclerotic tissue HS.
For the investigation, six samples were used per group. Each point represents a sample. The score is calculated from the cell type abundances by taking the logarithm of the expression of the genes specific to the cell types.
(**A**) Apoptosis score – A decreased apoptosis score is present in the sclerosis without any significant differences. (**B)** Autophagy score – A decreased oligodendrocytes score is detectable in the sclerosis. However, there are no significant differences. (**C)** Neuronal connectivity score – As expected, this score is higher in noHS. But the difference is not statistically significant. (**D**) Transmitter release score – The transmitter release score is increased in noHS since there is no neuronal cell death. The difference is not significant.
